# Uncanny sociocultural categories

**DOI:** 10.3389/fpsyg.2014.01456

**Published:** 2015-01-21

**Authors:** Jordan R. Schoenherr, Tyler J. Burleigh

**Affiliations:** ^1^Department of Psychology, Carleton UniversityOttawa, ON, Canada; ^2^Department of Psychology, University of GuelphGuelph, ON, Canada

**Keywords:** uncanny valley, cultural artifacts, categorical perception, categorization, social identity, cultural transmission

## Introduction

Humans are well adapted to their social environments. Experimental evidence suggests that humans are either born with, or quickly learn, the necessary affective and cognitive processes that allow them to recognize others, and to understand their mental states and social behavior. Mori's (1970) proposal of an uncanny valley, which describes affective response as a function of distance from a human category defined by morphological and behavioral features (i.e., human likeness), appears to be a sensible extension of these ideas. Following Mori's initial proposal, the uncanny valley has largely been considered in the context of cultural artifacts such as robotics, prosthetics, toys, and puppets. He associated “healthy people” with the greatest level of familiarity and positive affect, prosthetic hands and corpses with a global negative affective maximum, and bunraku puppets and humanoid robots with intermediate levels of familiarity and positive affect. It is important to note that these cultural artifacts represent the most contemporary features of human societies. The uncanny valley likely depends on extensions of prepotent responses to stimuli via general learning mechanisms (e.g., face recognition; Haxby et al., 2002; Sperber and Hirschfeld, 2004). Empirical studies of the uncanny valley have just begun to explore the authenticity of Mori's proposal.

Contemporary studies examining the uncanny valley hypothesis have drawn heavily on the psychological literature to explain these phenomena. The shift from an account of the uncanny valley based on a dimension of human-likeness to that of categorization and frequency-based exposure (Burleigh and Schoenherr, 2014) suggests that classes of cultural artifacts might provide evidence for the ubiquity of phenomena across cultures and within human history. These social representations can become external representations available to all members of a human group and can thereby increase familiarity and anchor human judgments (Moscovici, 1981). Supporting Mori's initial claim, negative responses are the result of a lack of familiarity (e.g., Burleigh and Schoenherr, 2014) that emerge over the course of development (Lewkowicz and Ghazanfar, 2012) as humans' affective systems have yet to adapt to these artifacts. If the uncanny valley does have a general cognitive basis, then evidence from affective, behavioral, and cognitive paradigms should exist both across cultures as well as within human history. These social representations will consequently affect observers' judgments.

### Human and non-human classification

#### Folktaxonomies and covert categories

A particularly compelling source of evidence for the uncanny valley comes from research into folktaxonomies. When we encounter an organism, our knowledge of folkbiological categories can cause us to classify stimuli in terms of a species (e.g., “fish”) or an ecological niche (e.g., “aquatic habitat”) that is available within a folktaxonomic structure. While the preferred level of categorization within these taxonomies differs between cultures (e.g., Rosch et al., 1976; Medin et al., 1997) and expertise (Tanaka and Taylor, 1991; Medin et al., 1997), such taxonomies form the basis for all judgements of category membership. In the context of this work, we suggest that cognitive anthropological research on folktaxonomies has revealed uncanny valley-like phenomena in the form of “covert categories”–categories that cannot be readily placed into a taxonomical structure (e.g., octopus). Covert categories are cognitively isolated from other ontological categories (Berlin, 1974; Atran, 1983). For instance, informants might be able to identify a number of basic-level properties of an octopus, and yet be unable to associate it with a superordinate category (e.g., “fish”). In Berlin et al.'s (1968) study of Tzetal Mayans' folktaxonomies, they found numerous covert groups that did not fall into any of the major lifeform categories (this term is used in the anthropological literature but is equivalent to the superordinate level in the psychological literature; c.f., Brown, 1974). These categories are also associated with aversive responses, such as food prohibitions (Douglas, 1966/2002; Sperber, 1996). For example, Henrich and Henrich (2010) observed that the ambiguity in classifying an octopus as a “fish” or “non-fish” was associated with a food taboo. Douglas (1957) also observed similar outcomes with other ambiguous animals, like the flying squirrel. Such responses to categorically ambiguous stimuli are consistent with the uncanny valley hypothesis.

#### Anomalies: gods and monsters

Related to covert categories is the human concern with biological anomalies, gods, and monsters evidenced throughout human history. In many cases, covert categories might be the basis for these ontological categories. As Sperber (1996) has pointed out in his discussion of hybrids and monsters, such entities tend to combine features from disparate categories; ranging from the addition of a single feature, such as *unicorns* which are horses with a horn, to more elaborate amalgams that combine multiple features, such as the *Minotaur* and *Manticore* which share features from human and animal categories. Representative of the cross-cultural trend are Japanese mythological creatures such as *Tsuchigumo*, a creature that has the face of a demon, the legs of a spider, and the body of a tiger.

Such mythological creatures might reflect social representations that are the products of cultural transmission. This is evidenced by Mayor's (2001, 2005) study of how ancient peoples classified fossilized creatures. Specifically, Mayor noted that ancient peoples appear to have understood fossils by assimilating them into extant ontological categories. In the absence of a clear understanding the process of fossilization, they would have perceived fossils as the scattered bones of what were believed to be living species. For instance, Mayor (2001) claims that when the fossilized remains of a protoceratops were encountered, in an attempt to account for the presence of these bones, ancients inferred the existence of griffons. Support for her argument is taken from the co-localization of fossils and reported observation of these creatures in ancient times. To ancients, these mythical creatures represented fearful stimuli that were associated with distant unknown territories inhabited by uncanny creatures.

Whereas fossil remains and unexplained natural events might help to explain the origin of mythological creatures (Mayor, 2001; Kaplan, 2012), along with responses such as fear and anxiety (also see Asma, 2009), the mechanisms that support the retention of these creatures in the modern mind requires further explanation (e.g., Barrett and Keil, 1996). Similar to the uncanny valley studies investigating categorization, studies of religious beliefs assume that belief in such ontological categories is a result of humans using existing cognitive templates that have exceptional features (Boyer, 1993, 2001). A heightened sensitivity to these anomalous stimuli, or so-called “counterintuitive beliefs” (Boyer and Ramble, 2001; Atran and Norenzayan, 2004), could be a result of increases in negative affect associated with the uncanny valley. These results can be related back to the idea of distinctiveness in memory (e.g., Hunt and Worthen, 2006), wherein the dissimilarity of an item within a given context facilitates the encoding and retrieval of stimuli. The uncanny valley could also have implications for recall that facilitates the cultural transmission of knowledge. As in the case of food taboos, such a negative valence might reduce our willingness to interact with features of our environment, thereby further reducing our exposure to a range of stimuli. An item's distinctiveness in memory can thereby compound an initial aversive response. Modern equivalents are also evidenced in genetically modified organisms that antagonist interest groups have labeled “Frankenfoods,” such as the transgenic tomato, which has been engineered with a gene from the winter flounder that makes it tolerant to freezing temperatures. Such rhetorical devices are clearly predicated on a fear of novel hybrid organisms.

#### Human categories and out-group bias

A final form of cross-cultural evidence for uncanny valley-like phenomena is the perception of human groups. Much like some non-human categories, unfamiliar human groups might be construed as distinct species. For instance, Gil-White (2001) suggests that this could in fact be the case for ethnicities. Identifying a race as “sub-human” implicitly or explicitly can be understood in these terms. For instance, a low-frequency of exposure to out-group members, or explicitly transmitted out-group biases, could create negative affective responses to features associated with these individuals (for the effect of stimulus frequency on affect, see Zajonc, 1968; Bornstein, 1989). Though controversial, numerous studies have found evidence for implicit negative associations with minority groups (Greenwald et al., 2009), which can be contrasted against explicit biases to “lower” social classes, and castes associated with “untouchability,” contamination, and food taboos (Harper, 1964). Accounts of early explorers encountering new tribes and peoples for the first time are also consistent with this possibility (e.g., Hall, 1992). Prophylactic measures were taken when entering strange lands inhabited by others and ritual purification following contact was needed to “guard against… the magical arts of its inhabitants” (Frazer, 1922, p. 110; see also Douglas, 1966/2002). For these explorers, the people that they encountered were similar to them but the comparatively small differences in terms of physical and cultural variation produced strong negative affective responses. As Hall (1992) notes of travelers' tales, features of out-group members were sometimes even perceived to reflect a blending of human and non-human animal traits: “In the land of Indian there are men with dogs' heads.” (quoted in Newby, 1975, p. 17).

A large degree of variation is also observed in the number and nature of reified gender and sexual categories across societies. Early First Nations societies in North America often recognized three or more sexes or genders and their associated roles within the society (Herdt, 1994). In sharp contrast, the early definition of homosexuality as a disorder in North American psychiatry reflects a perceived “deviation” from socially defined categories (Zucker, 2005). In the context of the present review, the experiences of homosexual, bisexual, and transgendered persons could reflect their status as a covert category in contemporary North American society. For instance, as described in a report by the San Francisco Human Rights Commission (2011), the experiences of bisexuals include being “rendered invisible” and being seen as vectors for the spread of sexually-transmitted diseases. Similar claims could be made concerning the historic status of women in Western and Near Eastern societies. For example, the unitary gender structure in the mythology of Abrahamic religions that sees Eve created from Adam's rib, framed the female sex as a derivative of the male sex. When framed in terms of deviation from a male reference category, the uncanny valley might offer a plausible basis for explaining negative affect and discrimination toward women. While exposure to male and female exemplars should be present in a society with nearly equal frequency, sociocultural practices can limit the exposure that one sex and gender has to another. While frequency alone is unlikely to account for all sex and gender biases, inasmuch as it does make a contribution it would support the existence of an uncanny valley. An understanding of the conditions in which the uncanny valley occurs, and whether increased exposure to low-frequency or negative-valence categories buffers against it, could facilitate our understanding of intergroup conflicts, and how they can be minimized. The codification of third or multiple gender categories minimally suggests that this is possible.

## Concluding remarks

Considered individually, folkbiological categories, biological anomalies and monsters, as well as human categories represent individual cultural products of human categorization. Instead, we suggest that the uncanny valley might reflect a primary response to unfamiliar or covert categories. In the absence of having prior knowledge of an individual or group, the relative distinctiveness of a category, due to a lower frequency of exposure, will produce negative affect—an inversion of the mere-exposure effect. The deceptive simplicity of learning mechanisms can lead to important individual and social consequences. If the uncanny valley and its relation to negative affect is a result of frequency of exposure, then its amelioration can be facilitated by increasing the frequency of the target stimuli within the environment. The few studies that have considered the relationship between familiarity, discriminability, and affect (Cheetham et al., 2013, 2014; Burleigh and Schoenherr, 2014) need to be complimented with more research that systematically manipulates the features of the ontological categories used for comparison.

### Conflict of interest statement

The authors declare that the research was conducted in the absence of any commercial or financial relationships that could be construed as a potential conflict of interest.
